# Assessment of the Relationship between the Total Occlusal Area of the Human Permanent Upper First and Second Molars and the Robusticity of the Facial Skeleton in Sex-Different Cranial Samples of Homo Sapiens: A Preliminary Study

**DOI:** 10.3390/biology12040566

**Published:** 2023-04-07

**Authors:** Wioletta Nowaczewska, Katarzyna Górka, Agata Cieślik

**Affiliations:** 1 Department of Human Biology, University of Wrocław, S. Przybyszewskiego 63, 51-148 Wroclaw, Poland; 2 Department of Anthropology, L. Hirszfeld Institute of Immunology and Experimental Therapy, Polish Academy of Sciences, Podwale 75, 50-449 Wroclaw, Poland

**Keywords:** human cranial robusticity, total occlusal area, permanent upper molars, supraorbital regions, facial superstructures

## Abstract

**Simple Summary:**

So far, only a few studies have focused on the issue of the relationship between the size of the occlusal surface of human permanent upper first and second molars and facial robusticity, and in none of them was the area of this surface precisely measured. Additionally, in these studies the sex of the examined skulls was not taken into account. In our study, for the first time, a precise method for obtaining the values of the area of the occlusal surface of these two types of molars was used to assess the relationship mentioned above for adult male and female skulls of *Homo sapiens* from the 19th century (exhibiting a wide range of robusticity). For each specimen, the grades of massiveness of six regions of the facial skeleton and general facial robusticity were assessed. The results indicated a difference between male and female skulls, including the presence of a significant relationship between the analyzed traits, and most of them (except in relation to the robusticity of the trigone region of the frontal bone in male cranial samples) did not support the “localized-masticatory-stress hypothesis”, suggesting the importance of the occlusal area as the trait influencing the formation of robusticity of the human facial skeleton.

**Abstract:**

The aim of this study was to establish whether there is a significant relationship between the total occlusal area (TOCA) of two types of permanent upper molars (first—M^1^ and second—M^2^) and facial robusticity, as well as which of the examined facial regions indicate a relationship concerning the grade of their massiveness with the TOCA of analyzed molars in different sex adult *Homo sapiens* cranial samples. To obtain the values of the TOCA of the molars (n = 145), a morphometric method was performed based on the calibrated digital images of their occlusal surface using ImageJ software. The grades of the massiveness of six facial regions were assessed using qualitative scales of their expression, and an index of general facial robusticity was calculated. Two types of analyses were performed concerning standardized and non-standardized traits to the facial size, including Spearman’s/or Pearson’s correlations and partial rank correlations. The obtained results indicated the presence of a positive relationship between the relative TOCA of M^2^s and the relative general facial robusticity, as well as between the TOCA of both types of molars and the massiveness of trigone region of the facial skeleton in male crania. However, most of the obtained results were not consistent with the assumptions of the “localized masticatory stress hypothesis”.

## 1. Introduction

It has been indicated by Demes and Creel [1] that bite forces are related to two main factors: the force of the muscles belonging to the masticatory apparatus and the dental pressure (occlusal area). The area of the occlusal surface of the crown of the human permanent molars has been considered as positively correlated with the bite/masticatory forces (e.g., [2,3,4]). It has also been commonly accepted that posterior dentition during mastication creates biomechanical stressing forces directed upward into the bones of the facial skeleton ([5,6,7,8,9,10,11,12,13,14] among others). However, so far, only a few studies have focused on the issue of the relationship between the size of the occlusal surface of the crown of the permanent upper molars and the robusticity of the modern human facial skeleton and in none of them was the precise measure of the area of the molar’s occlusal surface used (see [3,4]). The importance of the size of the occlusal surface of the permanent upper first (M^1^) and second (M^2^) molars for the expression of some of the traits of the facial skeleton’s robusticity was suggested by Lahr [3] and Lahr and Wright [4] in a sample of modern adult human crania (including male and female crania together) derived from geographically different regions of the world. However, their research did not exclude the influence of sex on the robusticity of the examined features. Their observations were interpreted as suggesting an influence of the localized masticatory forces/stress on the expression of the non-metric cranial traits [3,4]. According to this view (“the biomechanical hypothesis”) the robusticity of these traits has been considered as potentially formed to resist the stress generated during biting/chewing, as well as reflecting the influence of masticatory stress on their development; facial robusticity has been considered a phenotypic response to a high level of masticatory stress which takes place during ontogeny [3,4]. It is worthwhile to stress that formation of the robusticity of the modern human facial skeleton and the roles of the many suggested factors potentially influencing this process (including masticator stress and other traits, e.g., climate, size of the cranium, lifestyle, phylogenetic processes) are still not fully known (e.g., [5,15,16,17,18,19,20]).

Thus, the first aim of our study is to establish if there is a significant, positive relationship between the total occlusal area of the permanent upper molars (M^1^ and M^2^—considered separately) and the general robusticity of the facial skeleton in samples of the examined adult human crania (males and females analyzed separately) derived from Africa and Australia from the 19th century. In this study, for the first time, the method allowing the precise assessment of the size of the occlusal surface of these upper molars (contrary to other studies—see [3,4]) is used for the analysis of the relationship between this trait and facial robusticity. The occurrence of a significant positive relationship between these traits will indicate the importance of masticatory stress for the formation of the facial robusticity and, thus, will support the “localized biomechanical stress hypothesis”. It will also suggest the high developmental plasticity of the facial skeleton in the period from the eruption of M^1^ (around 5.5–6.0 years of age) [21] to the later time, encompassing an eruption of M^2^ and the period of the function of both of these teeth (in the sense of chewing and biting) until the end of the development and growth of the facial skeleton. However, taking into account the observation that the current knowledge about the changes in the patterns of the maximum bite force in post-canine teeth during modern human cranial ontogeny is not complete (see [22]), it is difficult to predict whether the patterns of the examined relationships will be similar in males and females, and in the case of both types of molars—M^1^ and M^2^. 

The permanent molars are a relatively separate developmental module (genetically independent) from other tooth modules (see, e.g., [23,24,25]). The studies concerning twins indicated a strong genetic determination of the size and shape of the crown of permanent molars, but also the meaning of the environmental factors [26,27]. For example, the result of the shared pre- and post-natal-environment influence (established for twins) of the size of M1 ranged from 22 to 27% of the contribution to variation of the crown phenotype [28]. All these data show the complexity of the process leading to the formation of a specific tooth-crown phenotype [25]. The facial skeleton is the part of the skull showing a significantly greater developmental reaction (“sensitivity”) to the influence of environmental factors than that of human teeth; however, it is also a complex structure including several developmental/functional modules [29,30,31,32]. Although the complex pattern of the different factors (phylogenetic, epigenetic, and environmental—e.g., climate, type of diet, and nutritional deficiency) influencing the expression of the facial skeleton phenotype (in a varying way influencing the shape and size of its different structural parts) has been established, the knowledge about the interaction of these factors during its development and growth is still not complete (see [33,34,35]). 

In studies attempting to explain the variation observed in both Late Pleistocene and modern human’s facial robusticity, the facial robusticity was mainly assessed based on the examination of the presence of the non-metric traits described as cranial superstructures (in the sense of Weidenreich [36]), such as, e.g., brow ridges/supraorbital torus, malar tubercle or zygomaxillary tuberosity, and grades of their expression/massiveness ([3,4,15,37,38,39,40], among others). The suggested functional meaning of these superstructures understood as their role in reducing (resisting) the masticatory forces influencing the formation of these traits during the growth of the facial skeleton has been interpreted as reflecting the developmental phenotypic plasticity of modern humans’ faces [3,4,15]. However, it is important to mention that the meaning of the biomechanical masticatory forces/stresses (also in sense of the size of the occlusal surface of molars—the greater size of this surface—the stronger generated bite/masticatory forces) for the formation of the robusticity of the facial skeleton and its specific regions has not been fully explained so far [4,15]. For example, according to the prevailing view, the biomechanical factor has not been universally recognized as the main cause of the formation of the supraorbital torus or the massive supraorbital ridge in the representatives of *Homo sapiens*; the significance of the position of the facial skeleton in relation to the anterior part of the base of the neurocranium and the shape of the squama of the frontal bone have been suggested as the main traits influencing the formation of the massiveness of this cranial superstructure, e.g., [38,40,41,42,43,44,45], contrary to other views describing the supraorbital torus/ridge as mainly reflecting a counteraction for masticatory stress (e.g., [46,47]). Our knowledge about the model of the precise interactions between the structural elements of the human facial skeleton during its development and growth is far from complete (see, e.g., [22]). The results of the studies focused on the patterns of the stress distribution in the 3D finite-element models of human adult male skulls (constructed based on their CT scans) caused by the simulated occlusal loading of the permanent upper molars have shown the presence of strain trajectories in the facial skeleton in response to the generated molar loads which divided from the point located above the place of load application into two branches, medial and lateral (e.g., [7,9,14]). The first of these branches was spread towards the glabella [7], the second lateral branch was extended superiorly in the zygomatic bone towards its frontal process, as well as into its temporal process; the identification of these trajectories (functional buttresses) was possible because of the occurrence of the higher values of the stress in these regions than in others located nearest to them in the bones of the facial skeleton [7,9,14]. Although the above-mentioned studies concerned the crania of adult individuals, the observations described above suggest the possibility of influence of the strains generated during chewing/biting on the formation of the massiveness of specific regions of the facial skeleton during the time of the growth of the facial skeleton. However, so far, the relationship between the size of the occlusal surface of the upper molars and grades of the expression of the specific facial superstructures in modern humans has not been investigated. 

Thus, the second aim of this study is to establish the relationship between the total area of the occlusal surface of the two types of molars (M^1^ and M^2^) and the grades of the massiveness of the specific facial superstructures concerning the supraorbital region of the frontal bone, the area of the body of the zygomatic bone, frontal process of this bone, and the zygomatic process of the temporal bone, separately for adult male and female crania. The presence of a positive relationship between these traits or some of them will support the “localized biomechanical stress hypothesis”. 

## 2. Materials and Methods

### 2.1. Samples

In this study, two samples of human skulls of adult individuals from Africa and Australia (derived from 19th century) were used [48,49,50,51]. The African skulls (from Uganda) [48,50] are stored in the Department of Anthropology of the Ludwik Hirszfeld Institute of Immunology and Experimental Therapy, Polish Academy of Sciences, Wrocław (Poland). The Australian skulls belong to the cranial collections of the Department of Human Biology at the University of Wrocław (Poland) [49,51]. From these two collections, only skulls of adult individuals exhibiting at least one of the permanent upper two molars (first—M^1^ or second—M^2^) embedded in the alveolar bone and facial skeleton in a good state of preservation were selected (see Table 1). In this study, data concerning the traits of facial skeleton were collected from 81 crania: 28 female crania (African cranial sub-sample—n = 19; Australian cranial sub-sample—n = 9) and 53 male crania (African cranial sub-sample—n = 33; Australian cranial sub-sample—n = 20) (see Table 1).

The crania were classified into the adult category based on the criterion of the presence of the complete fusion of the spheno-occipital synchondrosis and/or a permanent third molar (including the traits indicating the pre-mortem presence of this tooth) and/or the occurrence of the advanced stages of obliteration of cranial-vault sutures (see [52,53]). The sex of the African individuals was assessed based on the non-metric cranial traits according to the methodology of Ferembach et al. [54]. To avoid circularity, the traits concerning the three regions, the glabella, arcus superciliaris, and zygomatic process of temporal bone, were excluded from this assessment. The sex of the Australian individuals was obtained from literature and is consistent with their real sex (see [49,51]). In order to increase the variability of the examined features (which maximizes the possibility of detecting the relationship between them) the sample of female crania (also the sample of male crania) included the African and Australian specimens.

### 2.2. The Total Occlusal Area

**Figure 1:**
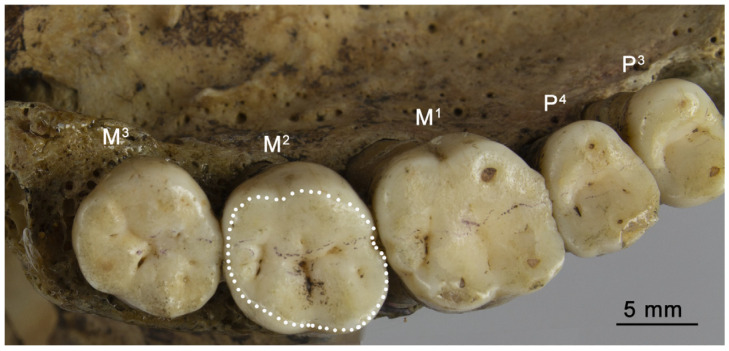
Occlusal view of permanent upper left teeth (P^3^, first premolar; P^4^, second premolar; M^1^, first molar; M^3^, third molar) with marked total occlusal area (TOCA) of the second molar (M^2^) (specimen no. 20).

The values of the total occlusal area (TOCA) (in mm^2^) were obtained by one of the authors (KG) from the images of the occlusal surface of the crowns of the left permanent upper molars (M^1^ and M^2^) according to the methodology used by Górka et al. [55,56]. The data about the total occlusal area were only collected for the upper right molars in cases of missing antimeres or when the upper left molars exhibited an inappropriate state of occlusal-area preservation related to the substantial enamel loss caused by caries or mechanical damage and/or a very high stage of crown wear. In this study, total occlusal area was obtained for 145 molars, including 69 M^1^s and 76 M^2^s (Table 1). Digital images (300 dpi) of the occlusal crown surfaces of the examined molars with a linear scale for calibration were taken using a Canon EOS 600 D camera fastened to a stand at a focal distance of 50 cm. Each skull was positioned in a way that the occlusal area of each of the photographed tooth was placed as parallel as possible to the camera lens. The scale was situated parallel to the camera lens and at the same height as the occlusal surface of the molar crown. ImageJ software [57] was used to measure the total occlusal surface area from the calibrated images; the perimeter of this surface was outlined with a minimum of 30 points using the polygon tool (see Figure 1).

In this study, the reliability of the method was examined by calculating the relative measurement error (RME) [RME = (Δ$\bar{x}$)/$\bar{x}$ × 100; Δx = Δx/√n] [45] on 20 randomly selected teeth measured three times at one-week intervals by KG. The average RME for the TOCA was 0.56%—lower than the 5% value, above which the method would be considered unreliable [58,59]. Consequently, the method was considered to be precise and repeatable.

It is worth mentioning that in the case of the method described above, the teeth exhibiting the severe crown wear were excluded from the samples of examined molars. Although the assessment of the stage of occlusal wear of the examined teeth was not directly related to the aims of this study, and this trait was not included in the statistical analysis, two scales of the stages of molar wear (including the modified Brothwell’s scale (see [60] p. 4) and the method proposed by Scott [61]) were used by one of the authors (WN) to obtain the data for this trait. These data were collected twice with an interval of 7 days between the first and second assessment—separately for each of the two methods. The Spearman’s rank correlation between the first and second assessment concerning both of these methods was significant and high (for modified Brothwell’s method, rs = 0.993, *p* = 0.000 and for Scott’s method, rs = 0.997, *p* = 0.000). The results of these analyses indicated high repeatability of these observations. The teeth with severe dentine exposition—over 14th stage in the case of the first of these scales (with a range from unworn molar crown to grade 18) and that over 32 points on the scale described by Scott [61] (with range from 4 to 40 points)—were not identified in examined samples of teeth (see Tables S1 and S2 for more details). Although the first of these scales was used by Mays et al. [60] for the study of the issue of the age at death assessment of the individuals from the British skeletal populations, results obtained by them included the data about the upper molar crown height reduction accompanying certain stages of dentine exposure (see [60], p. 7). Based on the results of their study, we could make a general estimation of the loss of the molars’ crown height related to the observed grades of their wear to be aware of the limitations of our research related to the problem of the importance of tooth crown height reduction for the TOCA values collected from the examined teeth. We describe and explain these limitations in “Discussion”. The second of two scales of dentine exposure in the occlusal surface of the crown of the permanent molars is commonly considered as more precise than the first, mentioned above, and universally used in the sense of being applicable to archaeological/historical human populations derived from geographically different areas, e.g., [62,63,64,65,66,67]. However, it is important to note that in our study these two scales were not used to assess the age at death of the examined specimens, but only to obtain the data about the stages of the wear of the crowns of the examined molars. The skulls with upper molars (M^1^ and/or M^2^) embedded in the alveolar process of maxilla used in this study belonged to adult individuals with a completely formed facial skeleton, thus the age at their death was not considered here as a factor influencing the formation of the massiveness of the examined regions of the facial skeleton. The age of the adult individuals does not directly influence the size of the TOCA of the permanent molars, which is completely formed relatively early in human ontogeny, e.g., [52,53,68]. However, molar crown height loss progresses with the age of the individual (e.g., [60]), but the inclusion in this study of only the upper molars with not very severe crown wear substantially reduces the indirect influence the age of individuals on the values of the TOCA of the examined teeth.

### 2.3. Traits of the Facial Skeleton 

**Figure 2:**
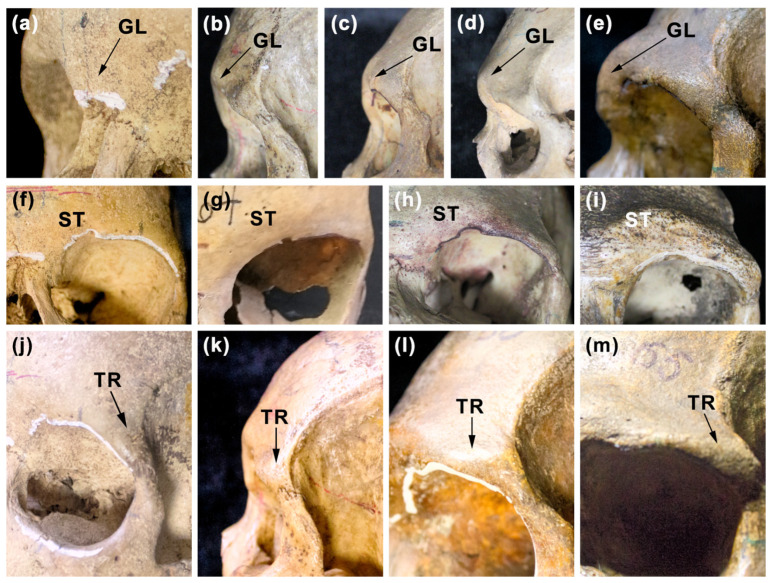
The supraorbital regions of the facial skeleton assessed in the examined human skulls in terms of their degree of massiveness expression: GL—glabella ((**a**) grade 1, flat area or minimal projection; (**b**) grade 2, slight prominence; (**c**) grade 3, moderate prominence; (**d**) grade 4, marked prominence; and (**e**) grade 5, the strongest, convex area and very well pronounced—loaf-shaped), ST—superciliary ridge ((**f**) grade 1, flat area or minimally projecting; (**g**) grade 2, clearly identifiable distinct unit; (**h**) grade 3, well pronounced; and (**i**) grade 4, anteriorly prominent area and very well pronounced), and TR—trigone ((**j**) grade 1, the area with a smooth or minimally salient surface; (**k**) grade 2, the area is raised in this region and extended anteriorly; (**l**) grade 3, the area is rounded, widened and prominent; and (**m**) grade 4, the area well developed and rounded with a rugged or non-rugged surface).

**Figure 3:**
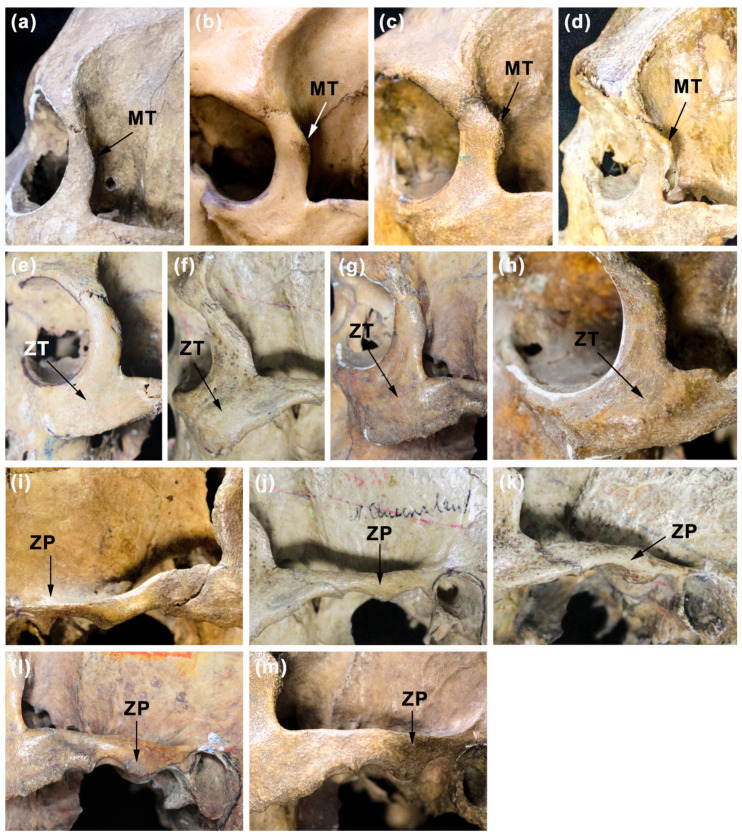
The regions of the facial skeleton assessed in the examined human skulls in terms of their degree of massiveness expression: MT—malar tubercle ((**a**) the margin is smooth, lack of bone elevation; (**b**) grade 2, a slight elevation of the posterior margin of this process (triangular in shape) is visible; (**c**) grade 3, the area of this elevation is greater than in the case of the second grade and rounded or a small convexity can be observed at this place; and (**d**) grade 4, the margin forms a well-developed process with two edges or one edge substantially raised upwards), ZT—zygomaxillary tuberosity ((**e**) grade 1, flat and smooth external surface of the malar bone; (**f**) grade 2, a small tubercle is visible; (**g**) grade 3, the tubercle is more pronounced and horizontally stretched; and (**h**) grade 4, a large tubercle in the form of a pronounced ridge located parallel to the inferior margin of this bone), and ZP—zygomatic process of temporal bone ((**i**) grade 1, a very thin process and low; (**j**) grade 2, process is thin and low; (**k**) grade 3, process is medium in the case of thickness and height; (**l**) grade 4, process is thick and high; and (**m**) grade 5, process is very thick and high).

The definitions of all the traits concerning the facial skeleton of the crania examined in this study are presented in Table 2 (see also Figure 2, Figure 3 and Figure S1).

These traits include the measure of the size of the facial skeleton (SFS) as the geo-metric mean of five metric traits (see Figure S1) of the human face encompassing measurements of its breadth, height, and length (it is a methodology often performed—see, e.g., [19,37,39,41,73]). The measurements used were selected from others that could potentially be included in the aforementioned measure, e.g., the height of the upper face (nasi-on-prosthion, M 48) or the total height of the face (nasion-gnathion, M 47) related to the size of the body of the mandible or the measurement of the bizygomatic breadth (zygion—zygion, M 45) (M—measurement defined by Martin and Saller [69], see [70]). Including these measurements requires a very good state of preservation of the facial skeleton.

Meeting this criterion in most of the examined skulls was not possible and would be associated with a significant reduction in the specimens in the analyzed samples. There was a lack of mandibles in many of these skulls, therefore the metric features of the mandibles were not included in the calculation of the measure of the facial skeleton size. 

The SFS was used to calculate the index of the relative general facial robusticity (R-RFS) and to standardize the TOCA of upper molars and the grades of the massiveness of examined facial regions to facial size (in order to eliminate the influence of the differences in the size of the facial skeleton among the examined specimens on analyzed relationships). The condition of the selection of the crania with all of the examined facial regions significantly reduced the number of specimens that were ultimately included in the analysis. It should be emphasized that the criterion for the selection of skulls was the presence of at least one permanent upper molar (out of two: M^1^ or M^2^) with a well-preserved crown and an appropriate state of preservation of the facial skeleton enabling the collection of the values of five metric features necessary to calculate the measure of its overall size and the assessment of the degree of massiveness of all from the six chosen facial regions. The grades of the robusticity of six regions of the facial skeleton (including: the glabella, superciliary ridge, trigone, malar tubercle, zygomaxillary tuberosity and the zygomatic process of temporal bone) were assessed for each of the selected skulls using the appropriate scales of the expression of their massiveness (see Table 2, Figure 2 and Figure 3). The grades of the robusticity for each of the examined non-metric traits were assessed two times with a one-week break for the skulls mentioned above and the results indicated the presence of small differences between the first and second assessment; these included a lack of any differences in the case of the traits, ST, ZT, and MT, to the observed difference in 1 in 20 (5%) of the examined skulls for the rest of the traits (including GL, TR and ZP), thus this methodology was considered acceptable and repeatable. All metric and non-metric traits of the facial skeleton of the crania examined in this study were collected by one of the researchers (WN). The cranial measurements were taken with sliding and spreading calipers with an accuracy of 0.5 mm.

### 2.4. Statistical Analysis 

All of the statistical analyses were performed separately for the M^1^s and M^2^s using the PAST (Paleontological Statistics software package for education and data analysis) [74] version 4.10, and 4.12 (the last of these versions was used for the analysis of the partial rank correlations) at the level of significance α = 0.05. To limit the influence of sex on the examined traits (in sense of the sex hormones shaping sexual dimorphism) (see, e.g., [75,76,77,78,79]) all of the statistical analyses were performed separately for samples of female and male specimens. To establish if there is a statistically significant relationship between the relative total occlusal area (TOCA/SFS) of the two types of molars (M^1^ and M^2^) and the index of the relative general facial robusticity (R-RFS) and the relative robusticity of each of the examined facial regions, Pearson’s correlation or Spearman’s rank correlations coefficients were calculated. The Spearman’s rank correlations were performed in all cases where the required conditions for the application of the Pearson’s correlation coefficients were not met (the condition of normality was assessed by using the Shapiro–Wilk W test). The partial rank correlation analyses were carried out for each of the six features reflecting the grades of the massiveness of the examined regions of the facial skeleton—separately for female crania and male crania (also separately for the groups concerning M^1^s and M^2^s). In total, 24 partial correlation analyses were performed (12 for female cranial samples and 12 for male cranial samples). In the case of this type of statistical analysis, the models included the massiveness of a considered area of the facial skeleton, the TOCA of examined molars (M^1^s or M^2^s), and the size of the facial skeleton (SFS). The partial rank correlations were performed to determine whether there was a statistically significant correlation between the TOCA of the two types of molars mentioned above and the grade of massiveness of the facial regions under examination, excluding the influence of the size of the facial skeleton on the examined relationship between these traits.

In cases of the cranial measurements taken from examined skulls to calculate the measure of the facial size (SFS), the intra-observer measurement error was analyzed using a paired t-test (the normality was assessed with a Shapiro–Wilk W test and the level of significance α = 0.05) based on the data obtained two times with a four-day interval for 20 randomly selected skulls.

## 3. Results

The obtained results concerning the metric traits used for the calculation of the SFS indicated the precision of these measurements (see Table S3). The descriptive statistics concerning the total occlusal area standardized to facial size (TOCA/SFS) of the permanent upper molars examined in this study, traits used to its calculation and the data about the proportions of the occurrence of the grades of massiveness of the facial regions for samples of females and males are presented in Table 3, Table 4 and Table S4.

Taking into account the first aim of this study, the obtained results of Spearman’s rank correlations indicated the presence of a statistically significant correlation between the TOCA/SFS of M^2^s and the index of relative facial robusticity (R-RFS) (positive and weak, rs = 0.33) only for a sample of male skulls (Table 5). No significant correlation between the TOCA/SFS of M^1^s and this index for female and male cranial samples was established (Table 5).

In the case of the second aim of this study, the results of Spearman’s rank correlation analyses indicated the presence of a significant correlation between the TOCA/SFS of M^1^s and only one of all (six) examined facial regions—the relative massiveness of the trigone region (TR/SFS; positive and weak, rs = 0.30) for male skulls and between the TOCA/SFS of M^1^s and only one trait—the relative massiveness of the glabellar region (GL/SFS; positive and moderate, rs = 0.58) for female skulls (Table 5). There were established the significant correlations between the TOCA/SFS of M^2^s and only the relative massiveness of two supraorbital facial regions: superciliary ridge (ST/SFS) positive and weak (rs = 0.30) and trigone (TR/SFS) positive and weak (rs = 0.35; higher in the case of the second of these traits) for a sample of male skulls. The results indicated the lack of the significant correlations between the TOCA/SFS of M^2^s and the relative massiveness of any examined facial regions for a sample of female skulls (Table 5).

In the case of the analyses concerning the non-standardized traits related to facial size, no significant Spearman’s rank and partial rank correlations were established between the massiveness of the examined facial regions and the TOCA of two types of molars, as well as between these traits and the facial size (SFS) for female cranial samples (Table 6 and Table 7). It is worthwhile to note that contrary to results of Spearman’s rank correlation between relative massiveness of GL and the relative TOCA of M^1^s (see Table 5) there were no significant Spearman’s rank correlation and partial rank correlation between massiveness of GL and the TOCA of M^1^s. It means that there was no significant relationship between these traits in examined female cranial sample. The above-mentioned difference is difficult to explain.

There were established a significant Spearman’s rank correlations between the massiveness of TR and the TOCA of two types of molars (positive and moderate) and also between the massiveness of GL, ST, ZT, and the TOCA of M^2^s (positive and weak) for male cranial samples (Table 8 and Table 9). 

However, in the case of the above-mentioned traits the significant partial rank correlations were established only between first of these traits (TR) and the TOCA of both types of examined molars (positive, weak for the TOCA of M^1^s and moderate for the TOCA of M^2^s) (Table 8 and Table 9). These results were generally congruent with that obtained for the Spearman’s rank correlation analyses between the grades of the robusticity of the facial regions standardized to facial size and the TOCA of molars standardized to facial size in male cranial samples (see Table 5). The one exception was the presence of a significant positive correlation between the ST/SFS and the TOCA/SFS of M^2^s. Although the obtained results indicated the presence of a significant Spearman’s rank correlation between grade of robusticity of the ST and the TOCA of M^2^s there was no significant partial rank correlation between these traits. It means that the relationship between these traits was not “true”, because the exclusion of the influence of facial size on the analyzed relationship caused the lack the significant partial correlation between these traits. Contrary to results obtained for female cranial samples, significant Spearman’s rank and partial rank correlations between the SFS and the TOCA of both types of molars (positive and moderate) for male cranial samples were established. However, this may have been due to the small number of female skulls. 

In summary, the obtained results concerning the second aim of this study indicated the presence of the significant partial rank correlations only between the grade of the massiveness of TR (from all six examined facial regions) and the TOCA of both types of molars—only in the case of male cranial samples. No “true relationship” (interpreted as not caused by the influence of the facial size) between traits reflecting the grades of robusticity of the examined facial regions and the TOCA of upper molars in female cranial samples was established.

## 4. Discussion

### 4.1. Relative TOCA of Upper Molars and the Relative General Facial Robusticity

Assuming the “localized masticatory stress hypothesis” (see [3,4,12]) is true, we should expect the presence of positive relationships between the TOCA/SFS of M^1^s and M^2^s and relative facial robusticity (R-RFS) for female and male cranial samples. The obtained results were only partially congruent with this assumption and most of them did not support this hypothesis. In this study, the significant positive correlation was established only between the TOCA/SFS of M^2^s (not the TOCA/SFS of M^1^s) and R-RFS in the case of male crania. It is worthwhile to note that this correlation was weak and there was no significant correlation between the traits mentioned above in any other cranial sample. However, the presence of this positive correlation can suggest that masticatory stress transmitted by the M^2^s up into the facial bones during the growth of the facial skeleton of males could stimulate the development of the massiveness of their facial superstructures. The results obtained for the samples of female skulls could be caused by their relatively small size (n = 21 for M^1^s, n = 26 for M^2^s); thus further investigation is needed, including a larger sample of female crania. However, we cannot exclude the possibility of the influence of the difference in the facial skeleton’s growth trajectory between females and males—the longer facial growth in males than in females determines the time of the influence of the masticatory stress transmitted through the upper teeth into the facial bones (also into regions of superstructures’ occurrence), which is longer in males than in females [80,81,82]. The lack of a relationship between the TOCA/SFS of M^1^s and the index of facial robusticity (R-RFS) established for the sample of male skulls is also difficult to explain. However, we cannot exclude the potential meaning of the pattern of the masticatory loading of the upper teeth during the period of the growth of the facial skeleton. Currently, our knowledge about this issue is still incomplete (see [22]). It has been observed by Edmonds and Glowacka [22], in their study of living individuals of different ages, that, in the group of juveniles without erupted permanent second molars, the bite forces were not highest at M1s but at the P4s (second premolars); juveniles with erupted M2s indicated the highest bite forces at M2s and the pattern of bite-force values, such as M1 > M2 > M3, occurred in subadults and adults. These observations show that, during the growth of the human face, the permanent second molars can be much more loaded than the permanent first molars by masticatory stresses, thus the “function” of M^2^ can be greater in the masticatory-stress transmission than M^1^, thus also in the stimulation of the development of facial superstructures. Detailed studies of the variation in bite forces of human teeth throughout the growth of the human face in geographically different populations would be very helpful for understanding the role of the size of the occlusal surface of the upper molars in the formation of facial superstructures.

### 4.2. TOCA of the Upper Molars and Robusticity of Examined Facial Regions

The results concerning the second aim of this study indicated on the presence of a positive correlation between the relative TOCA of both types of upper molars and the relative massiveness of trigone only in male cranial samples. The results also established significant positive Spearman’s rank correlation and partial rank correlations between the TOCA of these molars and the massiveness of the trigone in male crania exclusively. All these results suggest the occurrence of the positive relationship between the size of the occlusal area of M^1^s and M^2^s and grade of robusticity of the most lateral part of the supraorbital region of the facial skeleton and support the “biomechanical stress hypothesis”. Additionally the presence of the relationship mentioned above concerning the TOCA/SFS of M^2^s had most probably the greatest impact on the positive correlation established in this study between the TOCA/SFS of M^2^s and the index of relative facial robusticity in the case of the male cranial sample. Although the presence of the significant and positive correlation between the TOCA/SFS of M^2^s and ST/SFS in a male cranial sample can suggest the positive relationship between the TOCA of these molars and the grade of robusticity of the superciliary ridge with the exclusion of the influence of the facial size on this relationship, the obtained result of partial rank correlation between these traits indicated the lack of a relationship between these traits. Taking into account the lack of the significant Spearman’s rank correlation and partial rank correlation between the TOCA of M^1^s and the grade of the massiveness of GL region in the female cranial sample the presence of a significant and positive correlation between the TOCA/SFS of M^1^s and the GL/SFS established also for the same cranial sample is difficult to explain. No other significant relationships between the TOCA (and the TOCA/SFS) of both types of molars and the massiveness (and also the relative massiveness) of the examined facial regions (malar tubercle, zygomaxillary tuberosity, and zygomatic process of temporal bone) were established in female and male cranial samples. 

Thus, the most of the obtained results concerning the second aim of this study were not consistent with the assumption according to which the presence of a positive relationship between the TOCA (also the relative TOCA) of both types of molars (M^1^ and M^2^) and the grades of the massiveness (also the relative massiveness) of all of the examined non-metric facial traits should be expected. 

The theoretical assumption mentioned above was mainly based on the results of the study of Lahr [3], Lahr and Wright [4], and also based on the results of the studies concerning the 3-D finite models of the human cranium used to analyze masticatory stress/force distribution in the facial skeleton caused by occlusal loading of the maxillary molars (see, e.g., [7,9,14]). However, it is worth noting that the 3-D finite models of the crania used to analyze the distribution of the masticatory stress in facial bones concerned only male adult individuals who were not representative of Africans or Australians (see, e.g., [9,14]). The study of the distribution of the masticatory stress caused by occlusal loading of M^1^s or M^2^s in 3-D finite models of the skulls belonging to children and juvenile individuals from cranial samples could be helpful for understanding the meaning of biomechanical forces for shaping the facial superstructures. It is likely that the influence of the muscle of the masticatory apparatus, such as the masseter, was more important for stimulation of the development of some of the examined traits, e.g., massiveness of the zygomatic process of the temporal bone or zygomaxillary tuberosity or the temporal muscle also in the case of the first of these traits (see [83,84]); therefore further studies taking into account their size, cross-section, and location are necessary. 

The results of our study are only partially consistent with those obtained by Lahr [3]. Lahr [3] suggested the high importance of the occlusal area of M^1^s and also M^2^s for the development of the degree of expression of the supraorbital ridge and zygomaxillary tuberosity, and also the importance of the mesio-distal dimension of the M^2^s for the degree of robusticity of the zygomatic trigone in a sample of adult modern human crania derived from different parts of the world. However, the cranial sample used by Lahr [3] included male and female crania together, thus the influence of the sex on facial skeleton robusticity, its size, and also the size of the upper molars was not excluded (see, e.g., [75,78]). In Lahr’s [3] study the occlusal area of the examined molars was estimated (calculated using the standard measurements of the crowns of the molars—the mesio-distal and bucco-lingual diameters), traits, such as the grade of the development of the “supraorbital ridge”, the degree of the glabella expression, and the trait “zygomatic trigone”, concerned not only the trigonium (trigone) as the most lateral part of the supraorbital region of the frontal bone, but also the superior portion of the frontal process of the zygomatic bone—contrary to our study. All these observations could have influenced the differences between the results of our study and Lahr’s study. Lahr and Wright [4] used the bucco-lingual dimensions of M^1^s and M^2^s as the measure of the size of the occlusal surface of these molars and many cranial metric traits to assess the relationship between them and facial robusticity for unsexed cranial samples, including skulls of adult modern humans from geographically different regions of the world. They concluded that the close relationship established by them between palato-dental size and the robusticity of the facial skeleton supports the view suggesting the functional meaning of facial superstructures as structures resisting masticatory stress and the possibility of the influence of localized stress on the development of some of these structures (see [46,47,85]).

### 4.3. The Potential Influence of Diet and Non-Masticatory Activities on the Values of TOCA

#### 4.3.1. Meaning of Diet

The size and shape of the crown of the *Homo sapiens* permanent upper molars (including the value of their total occlusal area—TOCA) is fully formed at the moment of their complete development which already takes place before their eruption (e.g., for M^1^ on average at the age of 3.5 years—for females, and 3.6 years—for males, for M^2^ on average at the age of 6.9 years—for females, and 7.3 years for males [86]; more generally for M^1^ at the age range: 2.5–3.0 years and for M^2^ at the age range: 7.0–8.0 years [87]).

The size of a fully formed molar crown does not change during the growth of the facial skeleton, but it can be reduced by the progressive wear of the tooth crown occurring with the age of the individual (e.g., [88,89,90]). Two processes, such as attrition (tooth on tooth contact) and abrasion (the contact of the tooth crown with solid materials), are the main causes of this phenomenon [91,92,93,94]. When there is severe wear of the molar crown, the value of TOCA may differ from the original value of this trait (i.e., collected from the unworn tooth crown). Taking into account the bulbous shape of the crowns of the human permanent molars (e.g., [68,95]), it can be assumed that in the case of a severe degree of dentine exposure accompanied by a strong reduction in crown height, the TOCA value may be underestimated or overestimated in relation to the original value of this feature depending on the course of the occlusal wear plane. The horizontal occlusal wear plane (or with a relatively small angle of inclination) can be considered as related to underestimation of the TOCA value and the oblique occlusal wear plane (running obliquely, manifesting a clearly stronger degree of exposure of the dentine on one side of the occlusal surface of the crown—e.g., lingual in permanent upper molars) as related to overestimation of this trait. The occurrence of the first of these two types of the above-mentioned occlusal wear planes is often described as characteristic of hunter–gatherers and the second as specific to agriculturalists (e.g., [68,92,96,97,98]). 

According to Senator et al. [99], the skulls of Australians used in this study belonged to hunter–gatherers and those of Africans (from Uganda) most probably to shepherds and farmers. This means that the diet of these two groups of individuals may have differed primarily in terms of food hardness. Thus we can assume the presence of high abrasiveness of Australian hunter–gatherers’ diet—higher in comparison to the diet of Africans (see e.g., [62,95,100,101,102,103]. Unfortunately, we did not have detailed information on the diet of these two groups of examined individuals; in this study, the angle of course of the occlusal wear plane of the examined molars was not analyzed. It should also be noted that, in the case of the Australian sub-sample, it included crania of individuals from different areas of Australia (see [51]), which could be related to the greater diversity of their diet compared to individuals representing one population living in one specific area of Australia (see, e.g., [62]). In order to avoid large discrepancies between the actual TOCA value of the teeth examined in this study and the original value of this feature the molars with a very severe degree of crown wear were excluded from the analysis. The results of the assessment of the stage of dentine exposure of these teeth, which was performed using the modified Brothwell’s scale (including stages from 0 to 18) (see [60]) and the more precise Scott’s method (including scale from 4 to 40 points) [61], generally indicated the presence of a weak to moderate average stage of dentine exposure in the examined sub-samples of molars (for details see Tables S1 and S2). For example, in the case of the first from these two scales the results showed that the highest average stage of dentine exposure of M^1^s was obtained in Australian males (9.29) and females (7.86) compared to African males (6.84) and females (5.93). A similar pattern was observed for M^2^s, with the higher mean dentine exposure found for Australian males (5.42) and females (4.88) in comparison to African males (4.13) and females (3.33) (see Table S1). In addition, based on the results of Mays et al. [60] study indicating a loss of more than half the crown height of human permanent upper molars (M^1^ and M^2^) at the 12th stage of crown wear in the modified Brothwell’s scale, we checked how many of the examined molars showed a 12th or higher stage of crown wear. Of all the examined teeth, the 12th stage of this trait was present in only one M^1^ in Australian females and two molars (M^1^ and M^2^) in Australian males. A stage of this trait higher than 12th was only present in four Australian male molars (three M^1^s exhibited stage 14 of crown wear and one M^2^ exhibited stage 13 of this trait) (see Table S1). It can be assumed that in the case of these molars there was an increase in the risk of a greater discrepancy between their actual values of TOCA and the original values of this trait. The simplest solution in this situation would be to remove these teeth from the analysis. However, it could substantially reduce the number of molars examined in this study. 

The function of the upper molars (M^1^ and M^2^) related to masticatory activity is associated with the transfer of strains through these teeth to specific areas of the facial skeleton, which may affect the formation of their massiveness until the end of the facial skeleton growth process (potentially to 25 years—e.g., for posterior facial height—see [104]). This means that in a given individual, e.g., a male aged around 25 years, the average working time of M^1^ is already about 19 years and in the case of M^2^ 13 years (the average age of M^1^ eruption is 6 years and for M^2^—12 years—[68]; more precisely for African females: M^1^—5.4 years, M^2^—9.8 years and African males: M^1^—5.1 years; M^2^—10.5 years; Australian females: M^1^—6.5 years, M^2^—12.3 years and Australian males M^1^—6.7 years, M^2^—12.7 years [105,106]). The high degree of occlusal wear of the crowns of the first permanent molars has been identified even in young adult Australian Aborigines (see, e.g., [62,95]). Theoretically, the ideal solution for this problem mentioned here would be to obtain data on the TOCA of M^1^s and M^2^s during longitudinal studies of living individuals (in the early stages of the functioning of these teeth, first M^1^, then M^2^) also including collecting data on the massiveness of their facial skeleton at the stage of completion of its growth. However, this is impossible in the case of historical human populations.

#### 4.3.2. Meaning of Non-Masticatory Activities

Another factor that could potentially affect the analyzed relationships is extra-masticatory activities, i.e., related to the use of teeth to perform specific activities. This could generate additional stresses acting on certain areas of the facial skeleton and also contribute to the intensification and acceleration of wear of the molar crowns. However, it should be emphasized that in the case of Australian Aborigines, this type of activity was mainly associated with the use of the anterior dentition (e.g., [62,92,103,107]). It is believed that molars are the teeth least affected by non-masticating activities, which is why they are important in research on the type of diet (texture and abrasiveness of the food). In the case of this study, we did not have data on this type of activity of the examined groups of individuals and the analysis of changes observed on the teeth caused by extra-masticatory activities was not the purpose of this study.

### 4.4. Meaning of the Results and the Direction of Further Study

The results obtained in this study concerning the problem of the importance of the TOCA of upper permanent molars (first and second) for the formation of human facial robusticity indicated a lack of influence of this trait on the development of the massiveness of the examined facial superstructures, except for the lateral part of the supraorbital region of the frontal bone. Based on our results, the influence of localized masticatory stress related to the TOCA of both types of upper molars (in the sense of the transmission of masticatory strain to the facial bones) on the development of the robusticity of this region can be suggested. The phenotypic plasticity of the facial skeleton can be proposed as the potential biological mechanism explaining the “action” of this factor as it was considered in the case of the explanation of the formation of diet-driven differences in morphology of modern human crania (including the robusticity of the facial skeleton) (see [108]). However, without further analyses we cannot for certain exclude the possibility that the established positive significant correlations between the TOCA (also relative TOCA) of M^1^ and M^2^ and TR (also relative TR) and partial correlations between the TOCA of these molars and TR in male crania can be examples of covariances resulting from correlation with other traits not included in this study. Thus, the new analyses, including the other cranial traits which have been suggested as related to the expression of the massiveness of the supraorbital region of the facial skeleton, are needed. These traits can include, among others, the general shape of the neurocranium [15,38], the shape of the frontal bone [38,39,85], its position in the whole cranium, and the degree of facial retraction beneath the anterior part of the base of the cranium, e.g., [11,38]. In the case of the cranial samples, including the skulls of individuals of the same sex, the genetic factors seem to be most probably fundamental to the development of the traits of human facial robusticity because the expression of these traits has been interpreted as related to particular cranial constraints, such as cranial architecture and shape (see., [3,15,109]). The population-specific shape of the facial skeleton appears early in the cranial ontogeny ([81,110]), suggesting the meaning of the genetic factors as basic for determination of facial shape and, in this way, for facial robusticity. Making the assumption that the relationship between the TOCA (also relative TOCA) of upper molars and the massiveness (also relative massiveness) of the supraorbital area mentioned above established in this study is true in the sense described above (i.e., it is not only a “by-product” of the correlation of these traits with another feature), it would mean that mastication stress can be perceived as an additional factor influencing the formation of the massiveness of this area which is most probably mainly formed in response to genetic factors and sex (in the sense of the influence of sex hormones on facial skeleton morphology) e.g., [38,39].

## 5. Conclusions

This study demonstrated the lack of the relationship between the TOCA of two types of molars (M^1^ and M^2^) and most of the examined traits, reflecting the massiveness of six regions of the facial skeleton in male cranial samples. There was no relationship between these traits in female crania. Although the positive correlation between the relative TOCA of M^1^s and relative massiveness of the glabella in female cranial sample was established, there was no a significant partial rank correlation present between the TOCA of M^1^s and the grade of robusticity of the glabella in the same cranial sample.

Thus, the obtained results did not support the “localized masticatory stress hypothesis” with the exception of the presence of the positive relationship between the relative TOCA of M^2^s and the relative index of general facial robusticity and between the TOCA of both types of examined molars and the grade of the massiveness of the most lateral region of the supraorbital part of the facial skeleton (trigone) in male cranial samples. The results of this preliminary study suggest the presence of differences in functional meaning of the size of the occlusal surface of the permanent upper molars (M^1^s and M^2^s) for the formation of the massiveness of the supraorbital area of the facial skeleton between males and females. However, further study is needed, including a larger sample of specimens (especially female skulls with upper molars).

## Figures and Tables

**Table 1:** The number of upper first (M^1^) and second (M^2^) molars examined in this study; * both types of molars were present in the same skull.

Sample	M^1^	M^2^
All Females	21	26
Africans	14	18
Only M^1^ or M^2^ present	1	5
M^1^ and M^2^ *	13	13
Australians	7	8
Only M^1^ or M^2^ present	1	2
M^1^ and M^2^ *	6	6
All Males	48	50
Africans	31	31
Only M^1^ or M^2^ present	2	2
M^1^ and M^2^ *	29	29
Australians	17	19
Only M^1^ or M^2^ present	1	3
M^1^ and M^2^ *	16	16

**Table 2:** Description of the analyzed traits of the facial skeleton and the facial regions assessed in terms of the grades of their massiveness.

**Trait**	**Definition and Meaning**
SFS—measure of the size of the facial skeleton (mm)	Geometric mean of the following measurements (see Figure S1): nasion hormion length, nasal height (nasion—nasospinale chord, M 55), orbital height (M 52), outer biorbital width (frontomalare temporale—frontomalare temporale chord, M 43) and bimaxillary width (zygomaxillare—zygomaxillare chord, M 46). M—the measurements were taken according to Martin and Saller’s [69] definitions (see also [53,70]) except the nasion-hormion chord (the hormion point was placed at the crossing at a right angle to the sagittal midline with line runs through the point marked as the most posterior part of the vomer). The higher value of SFS means the greater size of the facial skeleton.
R-RFS—index of the relative robusticity of the facial skeleton (1/mm)	The sum of the grades of massiveness of all examined regions of the facial skeleton standardized to the size of the facial skeleton divided by the number of these regions, R-RFS = (GL/SFS + ST/SFS + TR/SFS + MT/SFS + ZT/SFS + ZP/SFS)/6. The higher the value of R-RFS, the greater the relative general massiveness of the facial skeleton.
**Facial region**	**Definition and the scale of its expression**
GL—glabella	The area at the glabella located above the nasal bones between the left and right arcus superciliaris (see, e.g., [71]). Scale according to Buikstra and Ubelaker [52] includes five grades from the weakest (1) to the strongest (5)—see Figure 2.
ST—superciliary ridge	The supraorbital region placed laterally from the glabella (see, e.g., [40]). Scale according to Nowaczewska et al. ([38], p. 112) includes four grades from the weakest (1) to the strongest (4)—see Figure 2.
TR—trigone	The most lateral portion of the supraorbital region (see, e.g., [71]). Scale according to Lahr [3] with modification concerning the exclusion of the assessment of the malar portion located nearest to the trigone includes four grades from the weakest (1) to the strongest (4)—see Figure 2.
MT—malar tubercle	The region of the posterior margin of the frontal process of the zygomatic (malar) bone, where the presence of spina of this process can be observed (see, e.g., [72]). Scale according to Piontek ([72], p. 83) with modification by adding the new grade between the original second and third grade includes four grades from the weakest (1) to the strongest (4)—see Figure 3.
ZT—zygomaxillary tuberosity	The region of the body of the zygomatic bone between its orbital and inferior margin where tuberosity or surface elevation can be observed. Scale according to Lahr ([3], p. 346–347) includes four grades from the weakest (1) to the strongest (4)—see Figure 3.
ZP—zygomatic process of temporal bone	The process of the temporal bone connecting this bone with the zygomatic bone (see, e.g., [53]). Scale according to Ferembach et al. [54] includes five grades from the weakest (1) to the strongest (5)—see Figure 3.
Relative robusticity of the examined facial regions (1/mm)	The massiveness of the examined facial region (GL, ST, TR, MT, ZT, ZP) in relation to the size of the facial skeleton (SFS): GL/SFS, ST/SFS, TR/SFS, MT/SFS, ZT/SFS, ZP/SFS. The higher value = the greater relative robusticity of the examined region of the facial skeleton.

**Table 3:** Descriptive statistics for the relative total occlusal area (TOCA/SFS) of the permanent upper molars and the traits used to its calculation (TOCA—total occlusal area; SFS—the size of the facial skeleton) presented for all samples of two types of molars (M^1^ and M^2^) used in this study.

Trait/Sample (Number of Specimens)	Minimum Value	Maximum Value	Mean	Standard Deviation
TOCA/SFS (mm)				
M^1^ Females (21)	1.32	1.96	1.58	0.16
M^1^ Males (48)	1.20	1.99	1.56	0.17
M^2^ Females (26)	1.09	1.76	1.41	0.19
M^2^ Males (50)	1.01	1.80	1.43	0.19
TOCA (mm^2^)				
M^1^ Females (21)	80.84	115.36	96.73	9.12
M^1^ Males (48)	75.22	133.51	100.98	12.42
M^2^ Females (26)	66.37	107.79	87.08	11.56
M^2^ Males (50)	63.70	121.33	92.99	13.67
SFS (mm)				
M^1^ Females (21)	57.29	65.92	61.18	2.35
M^1^ Males (48)	58.15	68.90	64.74	2.59
M^2^ Females (26)	58.35	65.92	61.76	2.29
M^2^ Males (50)	58.15	68.90	64.76	2.52

**Table 4:** The proportion (%) of the presence of the grades (from 1 to 4 or 5) of the massiveness of the examined regions of the facial skeleton (GL, glabella; ST, superciliary ridge; TR, trigone; MT, malar tubercle; ZT, zygomaxillary tuberosity; ZP, zygomatic process of temporal bone) in the analyzed cranial samples; N—maximal number (100%) of the specimens in the sample for which the data about the grade of massiveness for the examined facial regions were obtained.

Samples (N)	GL (1–5) %	ST (1–4) %	TR (1–4) %	MT (1–4) %	ZT (1–4) %	ZP (1–5) %
M^1^ Females (21)	1—76.19	1—71.43	1—85.71	1—52.38	1—90.48	1—38.10
	2—14.29	2—19.05	2—14.29	2—42.86	2—9.52	2—42.86
	3—9.52	3—4.76	3—0.00	3—4.76	3—0.00	3—19.05
	4—0.00	4—4.76	4—0.00	4—0.00	4—0.00	4—0.00
	5—0.00					5—0.00
M^1^ Males (48)	1—37.50	1—25.00	1—50.00	1—12.50	1—66.67	1—2.08
	2—29.17	2—33.33	2—27.08	2—50.00	2—14.58	2—20.83
	3—18.75	3—18.75	3—16.67	3—27.08	3—16.67	3—43.75
	4—6.25	4—22.92	4—6.25	4—10.42	4—2.08	4—25.00
	5—8.33					5—8.33
M^2^ Females (26)	1—80.77	1—65.38	1—73.08	1—53.85	1—80.77	1—26.92
	2—7.69	2—23.08	2—26.92	2—38.46	2—19.23	2—50.00
	3—11.54	3—7.69	3—0.00	3—3.85	3—0.00	3—23.08
	4—0.00	4—3.85	4—0.00	4—3.85	4—0.00	4—0.00
	5—0.00					5—0.00
M^2^ Males (50)	1—32.00	1—20.00	1—44.00	1—10.00	1—62.00	1—2.00
	2—28.00	2—34.00	2—26.00	2—50.00	2—14.00	2—18.00
	3—20.00	3—20.00	3—20.00	3—28.00	3—22.00	3—44.00
	4—10.00	4—26.00	4—10.00	4—12.00	4—2.00	4—28.00
	5—10.00					5—8.00

**Table 5:** Results of the correlation analysis between the relative total occlusal area (TOCA/SFS) of permanent upper molars and the traits of the facial skeleton analyzed in this study obtained for samples of female and male crania; rs—the value of Spearman’s rank correlation coefficient; r—the value of Pearson’s correlation coefficient.

Sample (N)/Traits	Correlation Coefficient	*p*-Value	Sample (N)/Traits	Correlation Coefficient	*p*-Value
M^1^ Females (21)			M^1^ Males (48)		
R-RFS	r = 0.28	0.218	R-RFS	rs = 0.26	0.076
GL/SFS	rs = 0.58	0.006 *	GL/SFS	rs = 0.17	0.243
ST/SFS	rs = 0.39	0.080	ST/SFS	rs = 0.22	0.128
TR/SFS	rs = 0.35	0.115	TR/SFS	rs = 0.30	0.035 *
MT/SFS	rs = 0.24	0.286	MT/SFS	rs = −0.06	0.697
ZT/SFS	rs = 0.29	0.199	ZT/SFS	rs = 0.11	0.454
ZP/SFS	rs = −0.02	0.924	ZP/SFS	r = 0.18	0.211
M^2^ Females (26)			M^2^ Males (50)		
R-RFS	r = 0.33	0.103	R-RFS	rs = 0.33	0.021 *
GL/SFS	rs = 0.35	0.082	GL/SFS	rs = 0.22	0.131
ST/SFS	rs = 0.14	0.346	ST/SFS	rs = 0.30	0.037 *
TR/SFS	rs = 0.13	0.530	TR/SFS	rs = 0.35	0.013 *
MT/SFS	rs = 0.04	0.841	MT/SFS	rs = −0.20	0.173
ZT/SFS	rs = 0.23	0.257	ZT/SFS	rs = 0.10	0.509
ZP/SFS	rs = 0.19	0.346	ZP/SFS	rs = 0.12	0.395

* *p* < 0.05 indicating statistical significance.

**Table 6:** Results of Spearman’s rank correlation analysis (top) and partial rank correlation analysis (bottom) obtained for examined traits including the TOCA of M^1^s—female cranial sample.

	**GL**	**TOCA M^1^**	**SFS**
GL	-	0.43 (0.0503)	0.14 (0.547)
TOCA M^1^	0.44 (0.051)	-	−0.04 (0.858)
SFS	0.17 (0.461)	−0.11 (0.632)	-
	**ST**	**TOCA M^1^**	**SFS**
ST	-	0.17 (0.450)	0.22 (0.343)
TOCA M^1^	0.18 (0.427)	-	−0.04 (0.858)
SFS	0.23 (0.332)	−0.08 (0.729)	-
	**TR**	**TOCA M^1^**	**SFS**
TR	-	0.07 (0.772)	0.07 (0.772)
TOCA M^1^	0.07 (0.768)	-	−0.04 (0.858)
SFS	0.07 (0.768)	−0.05 (0.846)	-
	**MT**	**TOCA M^1^**	**SFS**
MT	-	0.16 (0.483)	0.16 (0.483)
TOCA M^1^	0.17 (0.471)	-	−0.04 (0.858)
SFS	0.17 (0.471)	−0.07 (0.771)	-
	**ZT**	**TOCA M^1^**	**SFS**
ZT	-	−0.08 (0.729)	−0.13 (0.563)
TOCA M^1^	−0.09 (0.729)	-	−0.04 (0.858)
SFS	−0.14 (0.563)	−0.05 (0.858)	-
	**ZP**	**TOCA M^1^**	**SFS**
ZP	-	−0.09 (0.713)	0.04 (0.871)
TOCA M^1^	−0.09 (0.725)	-	−0.04 (0.858)
SFS	0.03 (0.886)	−0.04 (0.872)	-

*p* < 0.05 statistical significance, the value of “*p*” is presented in the parenthesis; the abbreviations used are explained in Table 2.

**Table 7:** Results of Spearman’s rank correlation analysis (top) and partial rank correlation analysis (bottom) obtained for examined traits including the TOCA of M^2^s—female cranial sample.

	**GL**	**TOCA M^2^**	**SFS**
GL	-	0.38 (0.053)	0.21 (0.304)
TOCA M^2^	0.37 (0.071)	-	0.13 (0.526)
SFS	0.17 (0.405)	0.06 (0.793)	-
	**ST**	**TOCA M^2^**	**SFS**
ST	-	0.13 (0.536)	0.15 (0.469)
TOCA M^2^	0.11 (0.601)	-	0.13 (0.526)
SFS	0.13 (0.522)	0.11 (0.589)	-
	**TR**	**TOCA M^2^**	**SFS**
TR	-	0.11 (0.593)	0.03 (0.889)
TOCA M^2^	0.11 (0.611)	-	0.13 (0.526)
SFS	0.02 (0.944)	0.13 (0.542)	-
	**MT**	**TOCA M^2^**	**SFS**
MT	-	0.04 (0.847)	0.11 (0.582)
TOCA M^2^	0.03 (0.905)	-	0.13 (0.526)
SFS	0.11 (0.604)	0.13 (0.546)	-
	**ZT**	**TOCA M^2^**	**SFS**
ZT	-	0.18 (0.391)	0.03 (0.875)
TOCA M^2^	0.17 (0.408)	-	0.13 (0.526)
SFS	0.01 (0.963)	0.13 (0.547)	-
	**ZP**	**TOCA M^2^**	**SFS**
ZP	-	0.16 (0.434)	0.01 (0.980)
TOCA M^2^	0.16 (0.442)	-	0.13 (0.526)
SFS	−0.02 (0.939)	0.13 (0.532)	-

*p* < 0.05 statistical significance, the value of “*p*” is presented in the parenthesis; the abbreviations used are explained in Table 2.

**Table 8:** Results of Spearman’s rank correlation analysis (top) and partial rank correlation analysis (bottom) obtained for examined traits including the TOCA of M^1^s—male cranial sample.

	**GL**	**TOCA M^1^**	**SFS**
GL	-	0.23 (0.114)	0.21 (0.159)
TOCA M^1^	0.14 (0.335)	-	0.55 (0.000) *
SFS	0.10 (0.513)	0.53 (0.000) *	-
	**ST**	**TOCA M^1^**	**SFS**
ST	-	0.31 (0.032)	0.38 (0.007) *
TOCA M^1^	0.13 (0.391)	-	0.55 (0.000) *
SFS	0.27 (0.067)	0.49 (0.000) *	-
	**TR**	**TOCA M^1^**	**SFS**
TR	-	0.41 (0.004) *	0.31 (0.034) *
TOCA M^1^	0.30 (0.038) *	-	0.55 (0.000) *
SFS	0.11 (0.480)	0.49 (0.001) *	-
	**MT**	**TOCA M^1^**	**SFS**
MT	-	0.05 (0.744)	0.26 (0.071)
TOCA M^1^	−0.12 (0.422)	-	0.55 (0.000) *
SFS	0.28 (0.053)	0.56 (0.000) *	-
	**ZT**	**TOCA M^1^**	**SFS**
ZT	-	0.28 (0.056)	0.33 (0.020) *
TOCA M^1^	0.12 (0.424)	-	0.55 (0.000) *
SFS	0.23 (0.127)	0.51 (0.000) *	-
	**ZP**	**TOCA M^1^**	**SFS**
ZP	-	0.19 (0.187)	0.29 (0.043) *
TOCA M^1^	0.04 (0.790)	-	0.55 (0.000) *
SFS	0.23 (0.123)	0.53 0.000) *	-

*p* < 0.05 statistical significance is marked with asterisk, the value of “*p*” is presented in the parenthesis; the abbreviations used are explained in Table 2.

**Table 9:** Results of Spearman’s rank correlation analysis (top) and partial rank correlation analysis (bottom) obtained for examined traits including the TOCA of M^2^s—male cranial sample.

	**GL**	**TOCA M^2^**	**SFS**
GL	-	0.31 (0.030) *	0.15 (0.307)
TOCA M^2^	0.27 (0.057)	-	0.55 (0.000) *
SFS	−0.03 (0.855)	0.54 (0.000) *	-
	**ST**	**TOCA M^2^**	**SFS**
ST	-	0.38 (0.010) *	0.35 (0.013) *
TOCA M^2^	0.24 (0.095)	-	0.55 (0.000) *
SFS	0.18 (0.210)	0.48 (0.001) *	-
	**TR**	**TOCA M^2^**	**SFS**
TR	-	0.49 (0.000) *	0.24 (0.094)
TOCA M^2^	0.44 (0.002) *	-	0.55 (0.000) *
SFS	−0.04 (0.804)	0.51 (0.000) *	-
	**MT**	**TOCA M^2^**	**SFS**
MT	-	−0.03 (0.825)	0.21 (0.149)
TOCA M^2^	−0.18 (0.220)	-	0.55 (0.000) *
SFS	0.27 (0.062)	0.57 (0.000) *	-
	**ZT**	**TOCA M^2^**	**SFS**
ZT	-	0.30 (0.036) *	0.24 (0.094)
TOCA M^2^	0.21 (0.158)	-	0.55 (0.000) *
SFS	0.10 (0.516)	0.52 (0.000) *	-
	**ZP**	**TOCA M^2^**	**SFS**
ZP	-	0.26 (0.070)	0.24 (0.100)
TOCA M^2^	0.16 (0.275)	-	0.55 (0.000) *
SFS	0.12 (0.429)	0.52 (0.000) *	-

*p* < 0.05 statistical significance is marked with asterisk, the value of “*p*” is presented in the parenthesis; the abbreviations used are explained in Table 2.

## Data Availability

The datasets generated and analyzed during this study are available to researchers upon request. Requests for data should be made the first author via email.

## Supplementary Materials

The following supporting information can be downloaded at: https://www.mdpi.com/article/10.3390/biology12040566/s1, Figure S1: Anthropometric points and linear measurements used in this study to calculate the measure of the facial size. Four measurements of the facial skeleton (**a**): nasal height (M 55: nasion (n)—nasospinale (ns) chord); orbital height (M 52); outer biorbital width (M 43: frontomalare temporale (fmt)—frontomalare temporale (fmt) chord; bimaxillary width (M 46: zygomaxillare (zm)—zygomaxillare (zm) chord). The position of the hormion (ho) point (**b**) which was used to obtain the facial length (nasion—hormion chord) as the fifth measurement included in measure of the facial size. Table S1: Results of the assessment of the occlusal wear stages in examined molars using modified Brothwell’s scale; G—grade of occlusal wear; M^1^—permanent upper first molar; M^2^—permanent upper second molar; Table S2: Results of the assessment of the occlusal wear stages in examined molars using Scott’s scale; M^1^—permanent upper first molar, M^2^—permanent upper second molar; Table S3: The intra-observer error—statistical analyses concerning the cranial measurements; Table S4: Descriptive statistics for the measurements of facial skeleton used in this study to calculate the measure of the size of the facial skeleton presented for all samples of two types of molars (M^1^ and M^2^).
