# Supplementary material for: Assessment of the Relationship between the Total Occlusal Area of the Human Permanent Upper First and Second Molars and the Robusticity of the Facial Skeleton in Sex-Different Cranial Samples of Homo Sapiens: A Preliminary Study
Source: Biology (Basel). 2023 Apr 7;12(4):566. doi: 10.3390/biology12040566 (PMC10136266; doi:10.3390/biology12040566)
Supplement: Supplementary file 1 [file biology-12-00566-s001.zip › Supplementary_Material_TABLE S1.docx]

**Table S1.** Results of the assessment of the occlusal wear stages in examined molars using modified Brothwell’s scale; G - grade of occlusal wear; M^1^ – permanent upper first molar; M^2^ – permanent upper second molar.

| **Sample**  **(N)** | **G1**  **N/%** | **G2**  **N/%** | **G3**  **N/%** | **G4**  **N/%** | **G5**  **N/%** | **G6**  **N/%** | **G7**  **N/%** | **G8**  **N/%** | **G9**  **N/%** | **G10**  **N/%** | **G11**  **N/%** | **G12**  **N/%** | **G13**  **N/%** | **G14**  **N/%** | **G15-18**  **N/%** |
| --- | --- | --- | --- | --- | --- | --- | --- | --- | --- | --- | --- | --- | --- | --- | --- |
| **M^1^** |  |  |  |  |  |  |  |  |  |  |  |  |  |  |  |
| Female All  (21) | (-) | 1/4.76 | (-) | 5/23.81 | 1/4.76 | 5/23.81 | 3/14.29 | 2/9.52 | (-) | 1/4.76 | 2/9.52 | 1/4.76 | (-) | (-) | (-) |
| Female AF  (14) | (-) | 1/7.14 | (-) | 3/21.43 | 1/7.14 | 4/28.57 | 2/14.29 | 2/14.29 | (-) | 1/7.14 | (-) | (-) | (-) | (-) | (-) |
| Female AUS  (7) | (-) | (-) | (-) | 2/28.57 | (-) | 1/14.29 | 1/14.29 | (-) | (-) | (-) | 2/28.57 | 1/14.29 | (-) | (-) | (-) |
| **M^2^** |  |  |  |  |  |  |  |  |  |  |  |  |  |  |  |
| Female All  (26) | (-) | 6/23.08 | 11/42.31 | 3/11.54 | 2/7.69 | 2/7.69 | (-) | (-) | (-) | 2/7.69 | (-) | (-) | (-) | (-) | (-) |
| Female AF  (18) | (-) | 5/27.78 | 7/38.89 | 3/16.67 | 1/5.56 | 2/11.11 | (-) | (-) | (-) | (-) | (-) | (-) | (-) | (-) | (-) |
| Female AUS  (8) | (-) | (-) | 4/57.14 | (-) | 1/14.29 | (-) | (-) | (-) | (-) | 2/28.57 | (-) | (-) | (-) | (-) | (-) |
| **M^1^** |  |  |  |  |  |  |  |  |  |  |  |  |  |  |  |
| Male All  (48) | (-) | (-) | (-) | 4/8.33 | 7/14.58 | 8/16.67 | 7/14.58 | 7/14.58 | 1/2.08 | 7/14.58 | 3/6.25 | 1/2.08 | (-) | 3/6.25 | (-) |
| Male AF  (31) | (-) | (-) | (-) | 4/12.90 | 5/16.13 | 5/16.13 | 5/16.13 | 7/22.58 | 1/3.23 | 3/9.68 | 1/3.23 | (-) | (-) | (-) | (-) |
| Male AUS  (17) | (-) | (-) | (-) | (-) | 2/11.76 | 3/17.65 | 2/11.76 | (-) | (-) | 4/23.53 | 2/11.76 | 1/5.88 | (-) | 3/17.65 | (-) |
| **M^2^** |  |  |  |  |  |  |  |  |  |  |  |  |  |  |  |
| Male All  (50) | (-) | 7/14.00 | 17/34.00 | 6/12.00 | 6/12.00 | 6/12.00 | 1/ 2,00 | 3/6.00 | (-) | 2/4.00 | (-) | 1/2.00 | 1/2.00 | (-) | (-) |
| Male AF  (31) | (-) | 5/16.13 | 10/32.26 | 4/12.90 | 5/16.13 | 4/12.90 | 1/3.23 | 2/6.45 | (-) | (-) | (-) | (-) | (-) | (-) | (-) |
| Male AUS  (19) | (-) | 2/10.53 | 7/36.84 | 2/10.53 | 1/5.26 | 2/10.53 | (-) | 1/5.26 | (-) | 2/10.53 | (-) | 1/5.26 | 1/5.26 | (-) | (-) |
