# Supplementary material for: Assessment of the Relationship between the Total Occlusal Area of the Human Permanent Upper First and Second Molars and the Robusticity of the Facial Skeleton in Sex-Different Cranial Samples of Homo Sapiens: A Preliminary Study
Source: Biology (Basel). 2023 Apr 7;12(4):566. doi: 10.3390/biology12040566 (PMC10136266; doi:10.3390/biology12040566)
Supplement: Supplementary file 1 [file biology-12-00566-s001.zip › Supplementary_Material_TABLE S2.docx]

**Table S2.** Results of the assessment of the occlusal wear stages in examined molars using Scott’s scale; M^1^ – permanent upper first molar, M^2^ – permanent upper second molar.

| **Sample (N)** | **Range**  **(min. - max.)** | **Mean** |
| --- | --- | --- |
| **M^1^** |  |  |
| Female All (21) | 8-30 | 20.66 |
| Female AF (14) | 8-25 | 19.57 |
| Female AUS (7) | 17-30 | 22.86 |
| **M^2^** |  |  |
| Female All (26) | 8-26 | 15.73 |
| Female AF (18) | 8-21 | 15.44 |
| Female AUS (8) | 8-26 | 16.38 |
| **M^1^** |  |  |
| Male All (48) | 17-32 | 22.54 |
| Male AF (31) | 17-29 | 21.19 |
| Male AUS (17) | 19-32 | 25.00 |
| **M^2^** |  |  |
| Male All (50) | 8-32 | 16.82 |
| Male AF (31) | 8-23 | 15.77 |
| Male AUS (19) | 12-32 | 18.53 |
