# Supplementary material for: Assessment of the Relationship between the Total Occlusal Area of the Human Permanent Upper First and Second Molars and the Robusticity of the Facial Skeleton in Sex-Different Cranial Samples of Homo Sapiens: A Preliminary Study
Source: Biology (Basel). 2023 Apr 7;12(4):566. doi: 10.3390/biology12040566 (PMC10136266; doi:10.3390/biology12040566)
Supplement: Supplementary file 1 [file biology-12-00566-s001.zip › Supplementary_Material_TABLE S4.docx]

**Table S4**. Descriptive statistics for the measurements of facial skeleton used in this study to calculate the measure of the size of the facial skeleton presented for all samples of two types of molars (M^1^ and M^2^).

| **Traits/Sample**  **(number of specimens)** | **Minimum**  **Value** | **Maximum**  **Value** | **Mean** | **Standard**  **Deviation** |
| --- | --- | --- | --- | --- |
| **Length of the facial skeleton: n-ho (mm)** |  |  |  |  |
| M^1^ Females (21) | 58.00 | 70.00 | 64.98 | 3.27 |
| M^1^ Males (48) | 60.00 | 79.00 | 69.78 | 4.10 |
| M^2^ Females (26) | 58.00 | 74.00 | 65.56 | 3.71 |
| M^2^ Males (50) | 60.00 | 79.00 | 69.83 | 4.17 |
| **Nasal height: n-ns** **(mm)** |  |  |  |  |
| M^1^ Females (21) | 37.00 | 52.00 | 44.86 | 3.48 |
| M^1^ Males (48) | 40.00 | 55.00 | 48.19 | 4.09 |
| M^2^ Females (26) | 41.00 | 52.00 | 45.83 | 3.15 |
| M^2^ Males (50) | 40.00 | 55.00 | 48.28 | 4.00 |
| **Orbital height** **(mm)** |  |  |  |  |
| M^1^ Females (21) | 29.00 | 39.00 | 33.48 | 2.29 |
| M^1^ Males (48) | 29.50 | 37.00 | 33.62 | 2.09 |
| M^2^ Females (26) | 30.00 | 39.00 | 33.87 | 2.23 |
| M^2^ Males (50) | 29.50 | 37.00 | 33.58 | 2.01 |
| **Outer biorbital width: fmt-fmt (mm)** |  |  |  |  |
| M^1^ Females (21) | 95.00 | 108.00 | 100.57 | 3.09 |
| M^1^ Males (48) | 100.00 | 116.00 | 108.00 | 4.34 |
| M^2^ Females (26) | 95.00 | 108.00 | 101.00 | 3.10 |
| M^2^ Males (50) | 100.00 | 116.50 | 108.27 | 4.32 |
| **Bimaxillary width: zm-zm (mm)** |  |  |  |  |
| M^1^ Females (21) | 78.00 | 97.00 | 87.74 | 4.61 |
| M^1^ Males (48) | 85.00 | 103.00 | 93.63 | 4.24 |
| M^2^ Females (26) | 78.00 | 97.00 | 87.79 | 4.60 |
| M^2^ Males (50) | 85.00 | 103.00 | 93.42 | 4.35 |
