# Supplementary material for: Assessment of the Relationship between the Total Occlusal Area of the Human Permanent Upper First and Second Molars and the Robusticity of the Facial Skeleton in Sex-Different Cranial Samples of Homo Sapiens: A Preliminary Study
Source: Biology (Basel). 2023 Apr 7;12(4):566. doi: 10.3390/biology12040566 (PMC10136266; doi:10.3390/biology12040566)
Supplement: Supplementary file 1 [file biology-12-00566-s001.zip › Sypplementary_Material_TABLE S3.docx]

**Table S3.** The intra-observer error – statistical analyses concerning the cranial measurements.

| **Facial skeleton measurements** | **Test (t-test)**  **Statistic** | ***p*-value** |
| --- | --- | --- |
| n-ho | -0.568 | 0.577 |
| n-ns | 0.000 | 1.000 |
| orbital height | 1.453 | 0.163 |
| fmt-fmt | -1.453 | 0.163 |
| zm-zm | 0.000 | 1.000 |

*p* < 0.05.
