# Supplementary figures and images for: Assessment of the Relationship between the Total Occlusal Area of the Human Permanent Upper First and Second Molars and the Robusticity of the Facial Skeleton in Sex-Different Cranial Samples of Homo Sapiens: A Preliminary Study
Source: Biology (Basel). 2023 Apr 7;12(4):566. doi: 10.3390/biology12040566 (PMC10136266; doi:10.3390/biology12040566)

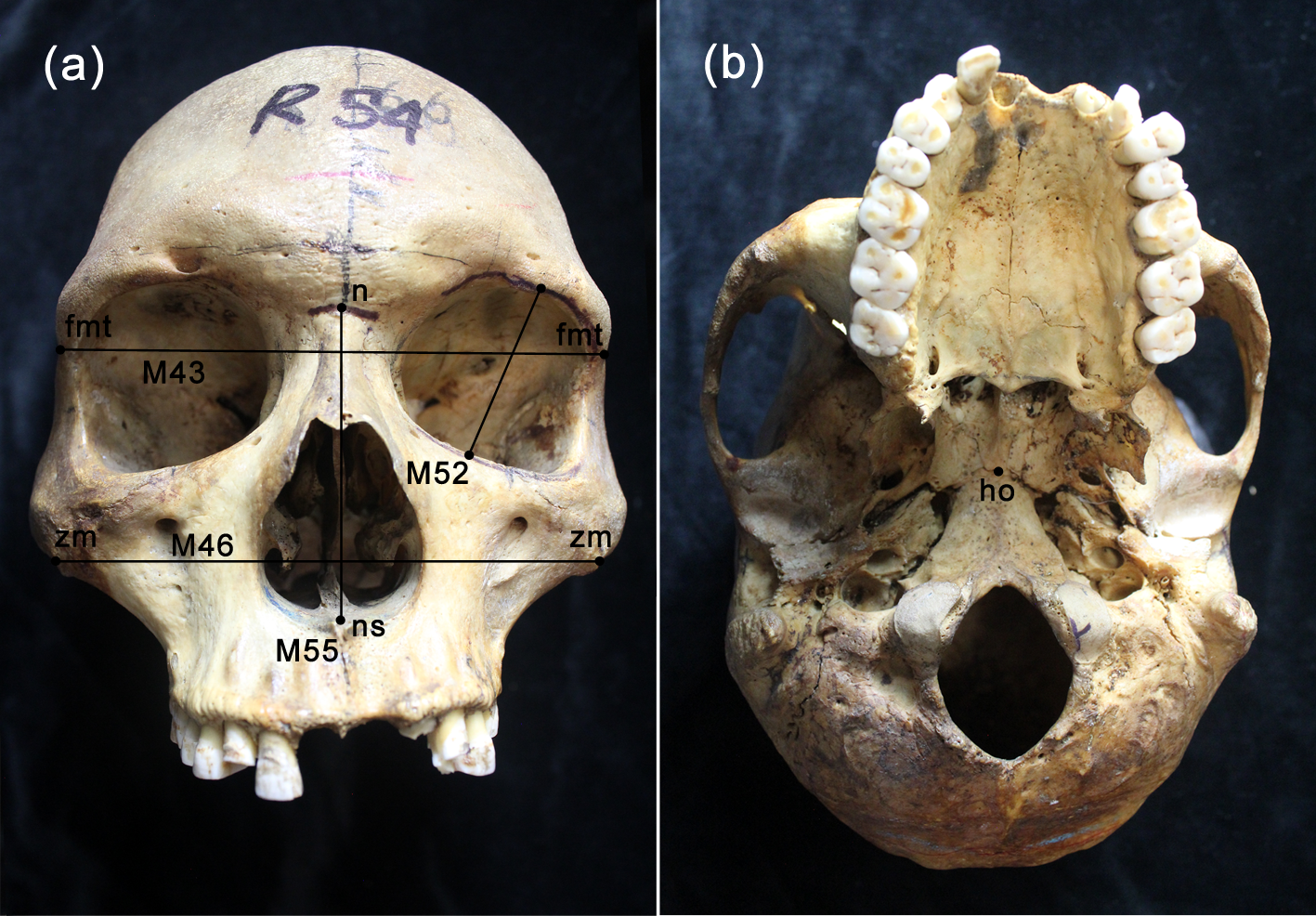

Supplement: Supplementary file 1 [file biology-12-00566-s001.zip › FIGURE_S1.tif]
